# Insulin signalling mediates the response to male-induced harm in female *Drosophila melanogaster*

**DOI:** 10.1038/srep30205

**Published:** 2016-07-26

**Authors:** Irem Sepil, Pau Carazo, Jennifer C. Perry, Stuart Wigby

**Affiliations:** 1 Edward Grey Institute, Department of Zoology, University of Oxford, Oxford, OX1 3PS, UK; 2 Instituto Cavanilles of Biodiversity and Evolutionary Biology, University of Valencia, Valencia, Spain; 3 Jesus College, University of Oxford, Turl Street, Oxford OX1 3DW, UK

## Abstract

Genetic manipulations in nutrient-sensing pathways are known to both extend lifespan and modify responses to environmental stressors (e.g., starvation, oxidative and thermal stresses), suggesting that similar mechanisms regulate lifespan and stress resistance. However, despite being a key factor reducing female lifespan and affecting female fitness, male-induced harm has rarely been considered as a stressor mediated by nutrient sensing pathways. We explored whether a lifespan-extending manipulation also modifies female resistance to male-induced harm. To do so, we used long-lived female *Drosophila melanogaster* that had their insulin signalling pathway downregulated by genetically ablating the median neurosecretory cells (mNSC). We varied the level of exposure to males for control and ablated females and tested for interacting effects on female lifespan and fitness. As expected, we found that lifespan significantly declined with exposure to males. However, mNSC-ablated females maintained significantly increased lifespan across all male exposure treatments. Furthermore, lifespan extension and relative fitness of mNSC-ablated females were maximized under intermediate exposure to males, and minimized under low and high exposure to males. Overall, our results suggest that wild-type levels of insulin signalling reduce female susceptibility to male-induced harm under intense sexual conflict, and may also protect females when mating opportunities are sub-optimally low.

Lifespan varies tremendously both among and within species, and is influenced by factors including sex, reproduction, and environmental stresses. Recent advances in molecular and physiological genetics have improved our understanding of the mechanisms by which lifespan is regulated. In recent studies, nutrition and nutrient-sensing pathways have emerged as highly conserved regulators of lifespan and of other major life history components such as growth, metabolism, stress resistance and fecundity^1^,^2^. Remarkably, mutations in the network formed by the insulin/insulin-like growth factor signalling (IIS) and target-of-rapamycin (TOR) signalling pathways have been found to extend lifespan in model organisms^3^,^4^,^5^. These pathways also modify the responses to common environmental stressors, such as starvation, oxidative and thermal stresses, and immune challenge. For instance, in *Caenorhabditis elegans* lifespan-extending manipulations also increase resistance to thermal and oxidative stresses^6^, whereas in *D. melanogaster* long-lived mutants exhibit reduced tolerance to heat and cold but increased resistance to starvation and oxidative stress^7^,^8^. Understanding the genetic pathways underlying stress resistance and susceptibility, and linking these pathways to lifespan regulation, remains an important challenge, particularly given the interest in applying this research to human lifespan^9^.

Male-induced harm, resulting from sexual conflict over mating, represents a stress imposed on females that can drastically reduce female lifespan^10^,^11^,^12^,^13^. At this point, we know that nutrition can influence female susceptibility to male-induced harm^14^,^15^, suggesting a potential role for nutrient-signalling pathways in mediating female responses to harm. However, despite a wealth of research on the lifespan-extending effects of nutrient-signalling pathways, few studies have considered how variation in mating activity affects lifespan in nutrient-sensing pathway mutants^16^. This is a significant gap not only because the lifespan-extending effects of these mutations should vary with mating activity through concomitant variation in male harm, but also because these same mutations that increase lifespan are known to decrease female re-mating rates^17^. It is therefore possible that the lifespan-extending effects of nutrient-signalling mutations result from their effects on reducing re-mating and thereby exposure to male harm. Because most studies of the genetics of ageing consider organisms with fixed and extreme (i.e., either very low or very high) mating regimes, potential interactions between mating rate, male-induced harm and lifespan extension through altered nutrient signalling remain poorly understood.

To address this gap, we measured female lifespan and reproductive traits in wild-type and long-lived transgenic *D. melanogaster* under varying levels of exposure to males. We used long-lived transgenic females in which the insulin-like-peptide (dilp)-producing median neurosecretory cells (mNSCs) are ablated late in development, by expressing a *UAS-reaper* (UAS-rpr) construct (an apoptosis-promoting factor) with a *InsP3-GAL4* driver (*UAS-rpr* > *InsP3GAL* females)^8^,^18^,^19^. The ablation of the mNSCs results in a significant decrease in *dilp2, dilp3* and *dilp5* expression in the fly, downregulating insulin signalling^19^. Hereon we refer to the *UAS-rpr* > *InsP3GAL* females as ‘ablated females’. These transgenic lines have been the target of multiple studies, which demonstrate that ablated females have increased lifespan and reduced egg-laying, both as virgins and after a brief (48 h) early-life exposure to males^7^,^8^,^20^,^21^,^22^. We have also previously shown that ablated females display reduced remating rates after a single copulation^17^. In this study, we exposed control and ablated females to four levels of male exposure throughout their life: 1 day followed by no further exposure (Treatment 0), 1 in every 8 days (Treatment 1/8), 1 in every 4 days (Treatment 1/4), or continuous exposure (Treatment 1). Intermediate levels of exposure to males are likely to maximize female fitness in wild-type flies^14^, because continuous exposure to males imposes survival costs on females^10^,^12^,^23^; whereas very low levels of male exposure likely result in sperm limitation^24^. Here, we explored whether varying levels of exposure to males affect the behaviour and fitness of control and ablated females differentially by measuring female lifespan, courtship and remating frequencies, fecundity, reproductive success, fertility and a population rate-sensitive estimate of fitness. We tested two sets of hypotheses:

(1) Lifespan extending manipulations of the insulin signalling pathway can modify the response to different environmental stressors in opposing ways^6^,^7^,^8^, hence we set out to examine both predictions for the response to male-induced harm, while under controlled environmental conditions. First, ablated females might be more resistant to male-induced harm compared with controls, as they are more resistant to certain environmental stressors (e.g., starvation)^7^,^8^. By this hypothesis, ablated females would display maximum lifespan-extension and fitness relative to controls under continuous exposure to males, and reduced or no differences under lower exposure. Alternatively, ablated females might be more susceptible to male-induced harm compared with controls, as they are more susceptible to some other environmental stressors (e.g., thermal stress)^7^ or because they may be frailer at older ages (e.g., as with *Caenorhabditis elegans* long-lived mutants^25^). By this hypothesis, the lifespan extension of ablated females would be minimized under continuous exposure to males, the treatment in which females are known to suffer costs of remating^10^,^12^,^13^,^23^.

(2) Under either of the above scenarios, we hypothesized that the greatest lifespan extension from mNSC ablation would coincide with the largest reproductive cost, due to the predicted trade-offs between lifespan and reproduction^26^.

## Results

### Lifespan

**Figure 1:**
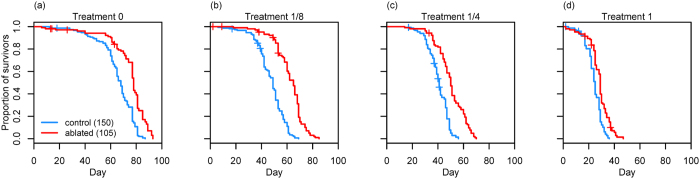
Survival of female flies with late ablation of the median neurosecretory cells (ablated females) and their genetic controls under varying levels of exposure to males: **(a)** one day of exposure followed by no exposure to males (Treatment 0); **(b)** one day of exposure in every eight days (Treatment 1/8); **(c)** one day of exposure in every four days (Treatment 1/4); **(d)** continuous exposure to males (Treatment 1). n_control_ = 150 and n_ablated_ = 105 in each male exposure treatment. The crosses indicate censoring.

Female lifespan declined with increased exposure to males for all genotypes (control females [Treatment 0 versus Treatment 1]: χ^2^_1_ = 347.28, *P* < 2.2e^−16^, hazard ratio = 271.873, 95% CI = 171.418– 431.19; ablated females [Treatment 0 versus Treatment 1]: χ^2^_1_ = 211.55, *P* < 2.2e^−16^, hazard ratio = 194.807, 95% CI = 115.038–329.891; see Supplementary Fig. S1). mNSC ablation significantly extended the lifespan of females within each male exposure treatment (Treatment 0: χ^2^_1_ = 53.084, *P* = 3.2e^−13^, hazard ratio = 0.360, 95% CI = 0.271–0.478; Treatment 1/8: χ^2^_1_ = 109.22, *P* < 2.2e^−16^, hazard ratio = 0.199, 95% CI = 0.145–0.274; Treatment 1/4: χ^2^_1_ = 74.988, *P* < 2.2e^−16^, hazard ratio = 0.274, 95% CI = 0.202 - 0.373; Treatment 1: χ^2^_1_ = 35.625, *P* = 2.4e^−09^, hazard ratio = 0.439, 95% CI = 0.333–0.579; Fig. 1). The median lifespan of ablated females in our 24-hour exposure treatment (treatment 0) was 78 (68–82), similar to values reported elsewhere (*UAS-rpr* > *InsP3GAL* median survival = 82 days^8^). When comparing the effect of the ablation against controls, we found a significant interaction with male exposure on lifespan (χ^2^_3_ = 14.463, *P* = 0.002). The lifespan extension was significantly greater for the ablated females of treatment 1/8 compared to the ablated females of treatment 0 (χ^2^_1_ = 7.622, *P* = 0.006, hazard ratio = 0.579, 95% CI = 0.393–0.853) and treatment 1 (χ^2^_1_ = 12.722, *P* = 0.0004, hazard ratio = 0.499, 95% CI = 0.339–0.736). Overall, mNSC ablation extended median lifespan under treatments 0, 1/8, 1/4 and 1 by 15%, 26%, 22% and 18% respectively (Supplementary Fig. S1).

### Courtship and mating behaviour

**Figure 2:**
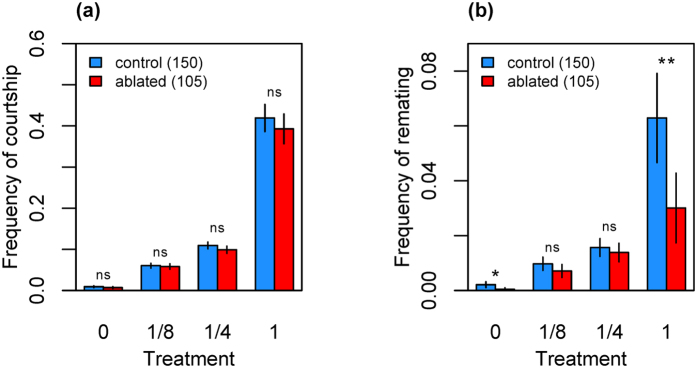
**(a)** Courtship and **(b)** remating frequency (±95% CI) of control and median neurosecretory cell (mNSC)-ablated females for each male exposure treatment. *P < 0.05; **P < 0.01; n.s., non-significant difference between control and ablated females. n_control_ = 150 and n_ablated_ = 105 in each male exposure treatment.

Both control and ablated females received similar rates of courtship when exposed to males: courtship frequencies did not differ between the control and ablated females for any treatment (Treatment 0: χ^2^_1_ = 1.684, *P* = 0.194; Treatment 1/8: χ^2^_1_ = 0.213, *P* = 0.644; Treatment 1/4: χ^2^_1_ = 2.941, *P* = 0.086; Treatment 1: χ^2^_1_ = 1.100, *P* = 0.294; Fig. 2a). However, compared with ablated females, control females of treatments 0 and 1 had significantly higher remating frequencies (Treatment 0: χ^2^_1_ = 4.774, *P* = 0.029; Treatment 1: χ^2^_1_ = 9.024, *P* = 0.003), whereas no difference was detected between the genotypes in treatments 1/8 and 1/4 (Treatment 1/8: χ^2^_1_ = 2.216, *P* = 0.137; Treatment 1/4: χ^2^_1_ = 0.578, *P* = 0.447; Fig. 2b).

### Fecundity, fertility and fitness

**Figure 3:**
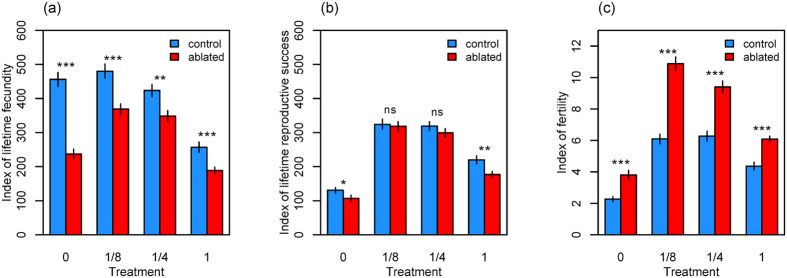
Indices of **(a)** lifetime fecundity, **(b)** lifetime reproductive success, and **(c)** lifetime fertility of control and mNSC-ablated females for each male exposure treatment. Asterisks indicate a significant difference (*P* < 0.05, permutation test) between control and ablated females within each male exposure treatment. Bars indicate 95% bootstrapped confidence intervals. *P < 0.05; **P < 0.01; ***P < 0.001; n.s., non-significant. See Table S1 for the number of vials retained for egg and offspring count every 4 days that were used for generating the indices.

Control females of each male exposure treatment had significantly higher early egg production (fecundity) than did ablated females, whereas no difference was detected later in life (see Supplementary Fig. S2). Consequently, control females of each male exposure treatment had significantly higher indices of lifetime fecundity (Treatment 0, Treatment 1/8, Treatment 1: *P* < 0.001; Treatment 1/4: *P* = 0.002; Fig. 3a). Control females had significantly higher early-life offspring production (reproductive success), but lower late-life reproductive success compared to ablated females of each male exposure treatment (Supplementary Fig. S2). This late life advantage in reproductive success for ablated females was most pronounced at intermediate levels of male exposure (treatments 1/8 and 1/4). Hence, control females of treatments 0 and 1 had significantly higher indices of lifetime reproductive success, whereas no difference was detected between control and ablated females in treatments 1/8 and 1/4 (Treatment 0: *P* = 0.037; Treatment 1/8: *P* = 0.792; Treatment 1/4: *P* = 0.231; Treatment 1: *P* = 0.003; Fig. 3b). Egg-to-adult offspring viability (fertility) was significantly higher in ablated females of each male exposure treatment compared with control females, for both maternal-age-specific offspring viability and indices of lifetime fertility (Treatment 0, Treatment 1/8, Treatment 1/4, Treatment 1: *P* < 0.001; Fig. 3c and Supplementary Fig. S2).

**Figure 4:**
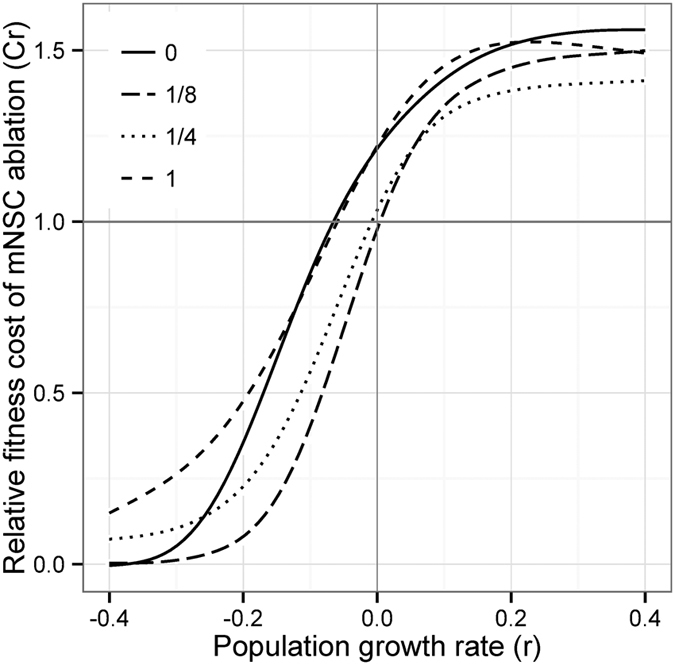
The relative fitness cost (*C*_*r*_) of mNSC ablation at different population growth rates (r) for each male exposure treatment. The grey horizontal line identifies relative fitness of 1; values above one indicate a fitness cost to mNSC ablation and values below one indicate a fitness benefit. The grey vertical line identifies a stable population with no population growth (*r* = 0). See Table S1 for the number of vials retained for offspring count every 4 days that were used for generating the relative fitness cost measures.

Finally, we computed a rate-sensitive fitness index to estimate population fitness under different rates of population growth. Overall, the relative fitness cost of mNSC ablation (*C*_*r*_) was estimated to be greatest in an expanding population (*r* > 0) for each male exposure treatment (Fig. 4). For the females of treatments 1/8 and 1/4, there was no cost of mNSC ablation in a stable population (*r* = 0), whereas the ablation was beneficial in a contracting population (*r* < 0). For the females of treatments 0 and 1, the benefit of mNSC ablation was only pronounced at smaller growth rates (r < −0.1) and it was costly in a stable population (*r* = 0).

## Discussion

We found that differing levels of exposure to males mediated the degree of lifespan extension achieved by ablated females relative to controls, suggesting that the insulin signalling pathway mediates female responses to male-induced harm. However, the change in lifespan extension did not follow a linear pattern with increasing exposure to males; instead, lifespan extension and fitness of ablated females were maximized under intermediate exposure to males, and minimized under both high and low exposure to males, implying that low- and high- mating environments are sub-optimal for long-lived females. The stress from male-induced harm in the high-mating environment is likely to be a result of intense sexual conflict over mating rates; hence, our results are consistent with the idea that ablated females are less resistant to the costs of male-induced harm compared with control females.

We have previously reported that ablated females remate less frequently than control females after a brief initial exposure to males^17^. Here, we find that this difference in remating occurs only at low (1 day) or high (continuous) exposure to males, whereas at intermediate levels of male exposure (1/8 and 1/4) ablated and control females remate at similar rates. Our mating observations were conducted on the morning after flies were first placed together within each 4-day cycle (see methods); for the 1-day exposure treatment, these observations therefore likely captured the remating of females that mated for the first time the day before. However, the most dramatic differences in remating were seen in the continuous exposure treatment, indicating that reduced remating rates of ablated females are not restricted to early life. The lack of difference in remating rates seen in the 1/8 and 1/4 treatments might be due to the transient nature of female post-mating refractoriness: although components of the male ejaculate reduce female sexual receptivity^27^, this effect is temporary in *D. melanogaster* and over the course of several days females deplete sperm reserves and regain virgin-like levels of receptivity^24^,^28^,^29^. The periods of 4 or 8 days between exposures to males may therefore have been sufficient for both control and ablated females to regain a substantial degree of sexual receptivity, resulting in a similar remating frequency.

We did not detect any significant differences in male courtship of ablated *versus* control females, suggesting that the differences in mating rates of females were largely female- rather than male-driven. Previous data show that IIS mediates female attractiveness, and that when given a choice males prefer to court females with high IIS^30^. However, in our experiments males were not given a choice of female types, and thus may have directed maximal courtship effort to the available females, irrespective of their IIS status. Harassment by males is harmful to females^31^, so reduced courtship could potentially add to the benefits of reduced IIS in mixed female IIS environments; however, this hypothesis remains to be directly tested.

Given that there are significant survival costs of remating for females when continuously exposed to males^10^,^12^,^13^,^23^, we might have predicted exaggerated lifespan differences in the continuous exposure treatment due to the combined effects of reduced remating and any lifespan-extending effects of mNSC ablation that are unrelated to mating rate. Nevertheless, we did not find this, and a potential explanation is that ablated females might be more sensitive to male-induced harm (e.g., more frail), which would reduce the potential survival benefits of reduced insulin signalling and remating under high mating conditions. Yet, by this hypothesis, we would predict the greatest lifespan extension and health of ablated females under the lowest male exposure – but again, this pattern was not observed. However, the result for the low male-exposure treatment–where after 1 day of exposure, females were isolated from males throughout life–should be interpreted with caution, as it likely reflects a biologically rare scenario. Both in our lab adapted population and in nature *Drosophila* mating rates are expected to be higher than one or two matings in a lifetime (an average of four to six times in nature^32^). Females in this group became sperm-depleted after day 20, producing no fertilized eggs despite living for an average of 78 days. Therefore, the decrease in lifespan extension for ablated females in this treatment might be related to increased investment in sexual signalling effort as they age^33^,^34^. Once females regain virgin-like levels of receptivity, they might switch to mate searching in order to obtain sperm. If ablated females are more susceptible to the costs of signalling receptivity, such as costs associated with the production of cuticular hydrocarbons (CHCs), or locomotion, we would see a reduction in lifespan extension, as observed here. Investigating changes in CHC profiles and locomotor activity with age, in relation to female mating status, will be necessary to provide further insight into the sexual signalling effort of wild-type and long-lived females.

Consistent with previous studies, ablated females laid eggs at reduced rates early in life, compared with controls^7^,^8^,^21^. This reduced early life fecundity may be a factor in their increased lifespan; for example, if high early-life fecundity induces greater survival costs than a lower but longer reproductive output^35^,^36^. However, our results do not fall into the simple pattern predicted by a Y-model of resource allocation between reproduction and lifespan^37^,^38^, because for ablated females we did not see relative increases in lifespan extension consistently accompanied by relative decreases in reproduction (i.e., in fecundity and fertility) across male exposure treatments. The largest difference in fecundity between ablated and control females occurred in the 1-day exposure treatment, but here we found only modest lifespan extension. Together, these data do not support the idea that lifespan extension due to reduced IIS reflects a straightforward trade-off with fecundity or mating. Yet, they suggest that the mechanisms that underlie insulin-signalling mediated lifespan extension are sensitive to the mating environment. Our results support the notion that lifespan and reproductive traits might be regulated in ways other than resource allocation, for example through reproductive signalling^39^. However, ablated females might display lifespan-reproduction trade-offs under more natural conditions that include food restriction or direct competition with other genotypes^40^,^41^,^42^.

Ablated females produced eggs with consistently higher egg-to-adult viability than controls across all male exposure treatments. To our knowledge no previous studies have measured fertility in long-lived transgenic *D. melanogaster* females, so it is unclear whether this is a common effect of reduced insulin signalling or an effect specific to mNSC ablation. Yet, the increased viability of eggs produced by ablated females partially (in the 1-day and continuous exposure treatments) or wholly (in the intermediate male exposure treatments) offsets the fecundity cost. Why the eggs of ablated females were more viable than controls is not known. A likely explanation is that ablated females divert resources from producing large number of eggs to producing higher quality eggs, with increased viability. A potential caveat would be if ablated females had higher fertility as a result of lower egg densities in the vials. However, this is unlikely since the largest viability difference between control and ablated females are detected later in life when egg densities are similar between genotypes, and we detect no differences in fertility early in life when fecundity is highest for the controls. Thus, when the densities of larvae are highest and most different between control and ablated females, we see no fertility differences.

Natural selection favours the product of reproductive ability and survival. We would therefore generally not expect to find mutations that can increase lifespan above wild-type levels without a concomitant reproductive cost. Yet, we apparently see this for ablated females in the 1/8 and 1/4 male exposure treatments in this study. However, because the rate of reproduction varied with age–with ablated females producing offspring at a reduced rate compared to controls early in life but an increased rate late in life–the fitness costs and benefits of mNSC ablation depend crucially on the population growth rate, with ablated females favoured when populations are declining, and controls favoured when populations are expanding. *D. melanogaster* populations in the lab and wild are likely to vary in population dynamics, cycling through periods of expansion and contraction, but in the long term will be stable or growing^13^,^43^. Thus it seems likely that the reproductive pattern of ablated females would be often disfavoured: as expected, wild-type would most commonly be the fittest phenotype. Moreover, the measures of survival and reproduction in this study were taken in a benign environment, with ad libitum food and no extrinsic sources of mortality. Even the population cage environment, to which the Dahomey population used in this study is adapted, is likely to be more exacting, with high densities at both the larval and adult stages and limited food resulting in intense competition. Moreover, because the population cages have high density, females are likely to experience intense and continual male harassment, with little opportunity for refuge, and little danger of sperm limitation. Thus, the continuous exposure treatment in our study likely represents the closest environment to which the flies are adapted–one characterized by high sexual conflict^44^– and, as expected, control females reproductively outperform ablated females in this treatment. Conversely, selection for reduced insulin signalling might occur in benign environments, for example where population density and sexual conflict are low, and/or in declining populations. A recent study investigated the genome-wide variation and differentiation patterns among three *D. melanogaster* populations collected along the North American east coast using sequences of DNA pools, and revealed that the variation indicated contracting winter population sizes in higher latitudes and population expansions in lower latitudes^45^. Moreover, lifespan, fecundity and mortality rates also vary predictably with latitude, such that northern populations are longer lived with reduced fecundity, whereas southern populations are shorter lived with high fecundity^46^. Furthermore, SNPs situated within the dilp3 gene, as well as other genes of the nutrient-sensing pathways, were highly differentiated between the southern and northern populations^45^. The question of whether the natural variants of these loci might directly underlie latitudinal differentiation in fitness-related traits is yet to be explored.

Recent decades have seen a revolution in our understanding of ageing mechanisms, and in our understanding of conflicts over mating between the sexes^44^. However, few studies have addressed how these fields can inform one another^47^. Here we have shown that mating rates and the genetic pathways that regulate ageing can interact to influence lifespan, and thus that these processes do not act in isolation. Our results raise the question of whether long-lived mutants might commonly be susceptible to male-induced harm under intense sexual conflict. To shed more light on this issue, future studies should consider how key lifespan regulating pathways evolve under differing levels of sexual conflict, and how genetic manipulations of female lifespan behave in populations with varying histories of male-harm.

## Materials and Methods

### Stocks and fly maintenance

We used a lab-adapted, outbred Dahomey wild-type stock, which has been maintained in large population cages with overlapping generations since 1970. A *white*^*Dahomey*^ stock was created by serially backcrossing *w*^*1118*^ into the Dahomey genetic background^7^. We ablated the mNSCs late in development in females by expressing *UAS-reaper* (*UAS-rpr*) under the control of the *InsP3-GAL4* (*InsP3GAL*) driver^8^. This was achieved by crossing *InsP3GAL* females with *UAS-rpr* males, and collecting the female progeny (*UAS-rpr* > *InsP3GAL*). *InsP3GAL* and *UAS-rpr* transgenic lines have been described and assayed elsewhere^8^ and are commonly used to genetically manipulate the IIS signalling pathway^48^. We backcrossed the *UAS-rpr* and *InsP3GAL* lines into the *white*^*Dahomey*^ genetic background for at least 5 generations prior to the experiment. We also used two sets of controls that are necessary and sufficient to account for any insertional mutagenic effects^7^,^8^,^48^. The first control *InsP3GAL* were the daughters of *InsP3GAL* females and *white*^*Dahomey*^males and controlled for the effects of the GAL4 driver (*InsP3GAL*/+). The second control *UAS-rpr* were the daughters of *white*^*Dahomey*^females and *UAS-rpr* males and controlled for the effects of the UAS insert (*UAS-rpr*/+). Males used in the experiment were all wild-type Dahomey. All flies were maintained at 25 °C on a 12:12 L:D cycle and fed Lewis medium^49^. Adult flies were maintained in 36 mL plastic vials containing Lewis medium supplemented with *ad libitum* live yeast grains or paste. Ablated and control flies were reared using a standard larval density method^50^, by placing approximately 200 eggs on 50 mL of food in 250 mL bottles. Virgins were collected on ice anaesthesia within 8h of eclosion and were aged in single-sex groups for 3–4 days before the experiment.

### Experimental design

Females were transferred to vials in groups of three and kept on a four-day cycle. On day 1 of the first cycle, three wild-type males were added to each vial for approximately 24 hours so that all the ablated and control females were mated. On day 2, following behavioural observations (described below), females were randomly allocated to one of four subsequent male exposure treatments: “0”, “1/8”, “1/4” or “1”. Treatment 0 females were not subsequently exposed to males; treatment 1/8 females were exposed to males on day 1 of every other 4-day cycle (i.e., every 8 days); treatment 1/4 females were exposed to males on day 1 of every cycle (i.e., every 4 days); and treatment 1 females were continuously exposed to males. 25 vials per male exposure treatment were set up for each control (*InsP3GAL* and *UAS-rpr*), and 35 vials per treatment were set up for the ablated females (*UAS-rpr* > *InsP3GAL*); therefore, each male exposure treatment had 150 control females (consisting of 75 *InsP3GAL* and 75 *UAS-rpr* females) and 105 ablated females at the beginning of the experiment. For each treatment, females were transferred to fresh vials on day 1 and day 2 of the four-day cycle, and vials from day 1 were retained to count eggs and score progeny (see below). For the continuous exposure treatment (treatment 1), males were transferred with females to fresh vials on day 2. For all other treatments, males were separated and held in single sex vials, while females were transferred to fresh vials. Flies were lightly anaesthetized using CO_2_ at each transfer. Experimental males were replaced with fresh 3–5 day old males on day 1 of every other cycle (i.e., every 8 days throughout the experiment) to limit the effects of male co-ageing on female life history traits. Replacement Dahomey males were obtained from stocks reared using the same standard larval density method as above. Female deaths were scored 5–6 days each week. To minimize density effects on mortality, two vials of the same treatment and genotype were merged when a single female was left in a vial owing to previous mortality or censoring. Hence, each vial had two or three females at all times and an equal sex ratio was maintained by adding the same number of males. Although the experimental design allowed us to have a large sample size, which is a necessity for conducting robust survival analyses, it prevented us from observing individual flies and calculating individual fitness measures. However, we were able to compute indices of lifetime fecundity, lifetime reproductive success and lifetime fertility to compare the fitness estimates of control and ablated females under different male exposure rates as detailed below in the statistical analyses section.

### Behavioural observations

On the evening of day 1, vials in which females were exposed to males were transferred to observation racks, where observations took place shortly after lights on (at 10am) on day 2 of every cycle. Observations were conducted between the 2^nd^ and 50^th^ day of the experiment, comprising 13 observation days, and lasted for approximately three hours, during which time vials were scanned continuously until every vial was scanned 3–6 times. We quantified the number of males courting females and the number of matings.

### Fecundity and fertility assays

On day 1 of every cycle females from each male exposure treatment were transferred into fresh vials supplemented with live yeast paste for approximately 24 hours. Females were then transferred into fresh vials on day 2 and the old vials were retained to count eggs and score progeny. Five vials per male exposure treatment were randomly selected for each control (*InsP3GAL* and *UAS-rpr*), whereas seven vials per male exposure treatment were selected for the ablated females (i.e., 17 vials in total) to estimate egg production (fecundity) in each cycle. Daily egg production was calculated as the number of eggs laid per female per 24 hours. The vials were then incubated at 25 °C for 11–13 days until adult eclosion. We used the same 17 vials per treatment to count the emerging offspring and estimate offspring production (reproductive success) and egg-to-adult offspring viability (fertility). Daily offspring production was calculated as the number of adult offspring emerging per female per 24 hours. Egg-to-adult offspring viability was calculated as the proportion of eggs laid from which adult offspring emerged.

### Statistical Analyses

We first tested whether the two control genotypes (*InsP3GAL* and *UAS-rpr*) had comparable responses. We used a Cox’s proportional hazards model implemented in the *survival* package in R^51^ to test whether male exposure treatments had a differential effect on female lifespan for the three genotypes (the two controls and the ablated). We used two *a priori* contrasts: (*i*) *InsP3GAL* control versus *UAS-rpr* control females, and (*ii*) ablated females versus merged control^48^. The initial model included ‘male exposure treatment’ and ‘genotype’ as factors and their interaction term. For both contrasts, we checked the global goodness-of-fit of the model based on the scaled Schoenfeld residuals and confirmed that the assumptions of proportional hazards were met (contrast i: χ^2^_7_ = 6.798, *P* = 0.45; contrast ii: χ^2^_7_ = 12.499, *P* = 0.085). These analyses revealed no detectable difference between the two control genotypes (*contrast i*: χ^2^_3_ = 5.159, *P* = 0.160), thus supporting the hypothesis that the male exposure treatment had similar effects on the lifespan of each control genotype (see Supplementary Fig. S3). We therefore merged the two control genotypes (*InsP3GAL* and *UAS-rpr*) as a single control for subsequent analyses.

We calculated an estimate for courtship and remating frequencies for control and ablated females of each male exposure treatment. This is to estimate how much courtship and remating the females were subject to throughout their lives. Courtship or remating frequency was defined as the proportion of females courted (or the proportion of females remating) on the observation day, corrected for male exposure differences between treatments^10^. Hence, the data was extrapolated to include the days in which the females of treatments 0, 1/8 and 1/4 were not exposed to males, as opposed to the females of treatments 1 that were continuously exposed to males. This correction was done as follows: For each treatment and genotype we summed the average number of females courted per vial on each observation day to calculate the total number of females courted (‘courtship’ total). For treatment 1, the ‘no courtship’ total is the number of females present on observation days that were not courted. For the other male exposure treatments, the ‘no courtship’ total is the number of females present on observation days that were not courted, plus the number of females present on the other days (when females were not exposed to males) until day 50 (the last day of behavioural observations). Likewise, for each treatment the ‘remating’ total is the number of females remating on the observation days. For treatment 1, the ‘not remating’ total is the number of females present on the observation days that did not remate. For the other male exposure treatments, the ‘not remating’ total is the number of females present on observation days that did not remate, plus the number of females present on the other days until day 50. We compared the courtship and remating frequencies of control and ablated females of each male exposure treatment using a 2 × 2 χ^2^ contingency analysis. Yates’ continuity correction was used if a cell had a count smaller than five.

We used two approaches to compare reproductive traits between control and ablated females within male exposure treatments. First, we calculated the age-specific egg production, offspring production, and egg-to-adult offspring viability of ablated and control females of each treatment and used Mann-Whitney-Wilcoxon tests to compare treatments, and Holm’s sequential Bonferroni correction to control for inflation in type 1 error rate due to multiple testing^52^. Mann-Whitney-Wilcoxon tests were used to overcome the underlying assumption of normality. Second, we calculated indices of lifetime fecundity, lifetime reproductive success and lifetime fertility^53^ to compare the fitness estimates of control and ablated females under different male exposure rates. Here, the means of age-specific egg production, offspring production and egg-to-adult offspring survival were multiplied by the age-specific proportion of survivors for each genotype in all male exposure treatments and summed to generate the indices; hence, a single value was obtained for control and ablated females of each treatment. To test for differences in the indices between control and ablated flies of each treatment, we used permutation tests with 1,000 iterations^54^. We present bootstrapped confidence intervals (based on 1,000 replicates) for indices of lifetime fecundity, lifetime reproductive success and lifetime fertility. Permutation tests and bootstrapping were conducted in Excel 2010 (Microsoft, Redmond, Washington, USA).

Finally, we adopted a population rate-sensitive estimate of fitness, which incorporates information on both the timing and number of offspring produced in life^13^, to determine the relative fitness costs of reduced insulin signalling for each male exposure treatment under different population growth rates. To do this, we calculated a rate-sensitive fitness measure *w*_pop_ for each treatment and genotype, from the fitness index developed by Charlesworth^55^. Values of intrinsic rates of population growth, *r,* were taken in the range of −0.4 to 0.4 as suggested for laboratory populations of *D. melanogaster*^56^. Values of *w*_pop_ were used to determine the relative fitness costs (*C*_*r*_) of mNSC ablation for different values of *r* defined as: *C*_*r*_ = *w*_pop cont._/*w*_pop abl_. We halved the offspring counts to reflect each female’s genetic contribution to its offspring^57^. All analyses were conducted using R v. 3.1.2^51^.

## Additional Information

**How to cite this article**: Sepil, I. *et al.* Insulin signalling mediates the response to male-induced harm in female *Drosophila melanogaster.* *Sci. Rep.* **6**, 30205; doi: 10.1038/srep30205 (2016).

## Supplementary Material

Supplementary Information
