# Supplementary material for: Insulin signalling mediates the response to male-induced harm in female Drosophila melanogaster
Source: Sci Rep. 2016 Jul 26;6:30205. doi: 10.1038/srep30205 (PMC4960482; doi:10.1038/srep30205)
Supplement: Supplementary Information [file srep30205-s1.doc]

**Electronic Supplementary Material**

**Insulin signalling mediates the response to male-induced harm in female *Drosophila melanogaster***

## Irem Sepil^1^, Pau Carazo^1,2^, Jennifer C Perry^1,3^, Stuart Wigby^1^

1. Edward Grey Institute, Department of Zoology, University of Oxford, Oxford, OX1 3PS, UK

2. Instituto Cavanilles of Biodiversity and Evolutionary Biology, University of Valencia, Valencia, Spain

3. Jesus College, University of Oxford, Turl Street, Oxford OX1 3DW, UK

**Figure S1 –** Median lifespan of female flies with late ablation of the median neurosecretory cells (ablated females) and their genetic controls for each male exposure treatments. Treatment 0 refers to one day exposure followed by no exposure to males; Treatment 1/8 refers to exposure to males one in every eight days; Treatment 1/4 refers to exposure to males one in every four days; Treatment 1 refers to continuous exposure to males. Bars indicate 25^th^ and 75^th^ percentile intervals.

**Figure S2 –** Age-specific egg production of control and mNSC-ablated females (±s.e.) under the male exposure treatments **(a)** one day followed by no exposure to males (Treatment 0); **(b)** one in every eight days (Treatment 1/8); **(c)** one in every four days (Treatment 1/4); **(d)** continuously exposed to males (Treatment 1). Age-specific offspring production of control and mNSC-ablated females (±s.e.) under the male exposure treatments **(e)** Treatment 0; **(f)** Treatment 1/8; **(g)** Treatment 1/4; **(h)** Treatment 1. Age-specific egg-to-adult offspring survival of control and mNSC-ablated females (±s.e.) under the male exposure treatments **(i)** Treatment 0; **(j)** Treatment 1/8; **(k)** Treatment 1/4; **(l)** Treatment 1. *p < 0.05; **p < 0.01; ***p < 0.001 for differences between control and ablated females corrected for multiple testing using Holm’s sequential Bonferroni correction.

**Figure S3 –** Median lifespan of female flies with late ablation of the median neurosecretory cells (*UAS-rpr>InsP3GAL*) and their genetic controls (*InsP3GAL/+* and *UAS-rpr*/+) for each male exposure treatment. Treatment 0 refers to one day exposure followed by no exposure to males; Treatment 1/8 refers to exposure to males one in every eight days; Treatment 1/4 refers to exposure to males one in every four days; Treatment 1 refers to continuous exposure to males. Bars indicate 25^th^ and 75^th^ percentile intervals.

**Table S1-** The number of vials that were retained every four days to calculate average egg and offspring production and egg-to-adult offspring survival of control and ablated females of each male exposure treatment. D2 (day 2) to D90 (day 90) indicates the days the vials were selected.

|  |  | **D2** | **D6** | **D10** | **D14** | **D18** | **D22** | **D26** | **D30** | **D34** | **D38** | **D42** | **D46** | **D50** | **D54** | **D58** | **D62** | **D66** | **D70** | **D74** | **D78** | **D82** | **D86** | **D90** |
| --- | --- | --- | --- | --- | --- | --- | --- | --- | --- | --- | --- | --- | --- | --- | --- | --- | --- | --- | --- | --- | --- | --- | --- | --- |
| Treatment  0 | n_control_ | 10 | 10 | 10 | 10 | 10 | 10 | 10 | 10 | 10 | 10 | 10 | 10 | 10 | 10 | 10 | 10 | 10 | 10 | 10 | 9 | 2 | 1 | 0 |
|  | n_ablated_ | 7 | 7 | 7 | 7 | 7 | 7 | 7 | 7 | 7 | 7 | 7 | 7 | 7 | 7 | 7 | 7 | 7 | 7 | 7 | 7 | 7 | 6 | 3 |
| Treatment 1/8 | n_control_ | 10 | 10 | 10 | 10 | 10 | 10 | 10 | 10 | 10 | 10 | 10 | 10 | 10 | 10 | 9 | 3 | 1 | 0 | 0 | 0 | 0 | 0 | 0 |
|  | n_ablated_ | 7 | 7 | 7 | 7 | 7 | 7 | 7 | 7 | 7 | 7 | 7 | 7 | 7 | 7 | 7 | 7 | 7 | 7 | 6 | 2 | 1 | 0 | 0 |
| Treatment 1/4 | n_control_ | 10 | 10 | 10 | 10 | 10 | 10 | 10 | 10 | 10 | 10 | 10 | 10 | 4 | 2 | 0 | 0 | 0 | 0 | 0 | 0 | 0 | 0 | 0 |
|  | n_ablated_ | 7 | 7 | 7 | 7 | 7 | 7 | 7 | 7 | 7 | 7 | 7 | 7 | 7 | 7 | 7 | 7 | 3 | 1 | 0 | 0 | 0 | 0 | 0 |
| Treatment  1 | n_control_ | 10 | 10 | 10 | 10 | 10 | 10 | 10 | 9 | 2 | 0 | 0 | 0 | 0 | 0 | 0 | 0 | 0 | 0 | 0 | 0 | 0 | 0 | 0 |
|  | n_ablated_ | 7 | 7 | 7 | 7 | 7 | 7 | 7 | 7 | 8 | 3 | 1 | 1 | 0 | 0 | 0 | 0 | 0 | 0 | 0 | 0 | 0 | 0 | 0 |
